# Precaution with standards: rethinking respiratory sensitization classification through the case of methyl methacrylate

**DOI:** 10.3389/ftox.2026.1813881

**Published:** 2026-05-15

**Authors:** Frank Faulhammer

**Affiliations:** BASF SE, Global Toxicology and Ecotoxicology, Ludwigshafen, Germany

**Keywords:** methyl methacrylate (MMA), new approach methodologies (NAMs), precautionary principle, regulatory toxicology, respiratory irritation, respiratory sensitization, specific inhalation challenge (SIC)

## 1 Introduction

Respiratory sensitization is among the most challenging hazard endpoints to classify, as the clinical evidence base is often sparse, heterogeneous, and variably documented. At the same time, regulatory toxicology is increasingly informed by mechanistic evidence from new approach methodologies (NAMs), which can provide insight into early key events relevant to respiratory sensitization pathways [e.g., KE1–KE3 in the MMA case study] (Faulhammer et al., 2026), and are being framed within evolving AOP-based conceptual models for respiratory sensitization (Hargitai et al., 2024). This raises a practical question for decision-makers: how should precaution be applied when human evidence is limited or methodologically constrained, while mechanistic data point towards an alternative mode of action?

The ongoing debate on whether methyl methacrylate (MMA) should be classified as a respiratory sensitizer—beyond its established classification as a respiratory irritant—illustrates this tension. In 2024, the EU Committee for Risk Assessment (RAC) reaffirmed MMA as a Category 1 respiratory sensitizer, relying primarily on six unpublished positive specific inhalation challenge (SIC) cases reported in Annex 5 of the 2021 opinion, three of which originated from the Finnish Institute of Occupational Health (FIOH), a specialized occupational medicine center (European Chemicals Agency, 2024). However, regulatory documentation indicates that several elements critical for interpreting SICs as evidence of immune-mediated respiratory sensitization—such as controlled non-irritant exposure conditions, substance specificity, and comprehensive clinical characterization—are limited or missing in these cases (European Chemicals Agency, 2024; Pemberton and Kimber, 2022). In parallel, available NAM evidence has been interpreted as inconsistent with a respiratory sensitization mode of action for MMA (Faulhammer et al., 2026).

From a regulatory perspective, RAC’s precautionary stance is understandable. Respiratory sensitization represents a serious and potentially irreversible health outcome, and CLP guidance allows classification on the basis of limited human evidence where uncertainty cannot be confidently resolved. The present Opinion does not challenge this protective intent. Rather, it examines whether the evidence underpinning the classification meets the interpretability and mechanistic plausibility expectations outlined in CLP Annex I and whether MMA’s known respiratory irritant properties provide a more plausible explanation for the observed SIC responses.

This Opinion argues for *“precaution with standards”*: maintaining a high level of protection while ensuring that evidence used for respiratory sensitization classification meets minimum quality criteria and remains mechanistically coherent. Using MMA as a case example, it highlights where documentation and interpretability thresholds are particularly critical and proposes a transparent approach for integrating data with NAM- and AOP-based mechanistic evidence within the CLP framework (European Union, 2008; European Chemicals Agency, 2024; Faulhammer et al., 2026; Pemberton and Kimber, 2022).

As an Opinion article, this contribution does not aim to provide a systematic review of all available evidence but focuses on key data relevant to the current regulatory interpretation of MMA.

## 2 What the SIC evidence for MMA does—and does not—support

RAC initially reviewed six unpublished SIC-positive cases but ultimately deemed only four of them relevant for MMA, excluding the cyanoacrylate/powder case and the PMMA dust sanding case (European Chemicals Agency, 2024). Yet, as documented in regulatory materials and secondary evaluations, key limitations reduce interpretability of these SICs as evidence for immune-mediated sensitization rather than irritation-driven bronchoconstriction (European Chemicals Agency, 2024; Pemberton and Kimber, 2022). Three cases were not clearly substance-specific: one involved a cyanoacrylate–acrylic powder mixture, another involved polymethyl methacrylate (PMMA) dust generated by sanding a hardened prosthesis, and a third used neat MMA brushed onto cardboard without exposure monitoring, which RAC nevertheless considered relevant (European Chemicals Agency, 2024). In the first two cases, the presence of mixed or particulate exposures precluded attribution of the observed responses to MMA alone. While cyanoacrylates and PMMA dust have been associated with occupational asthma and respiratory symptoms in clinical settings, these co-exposures do not permit substance-specific causal interpretation for MMA under CLP criteria, underscoring their limited value for respiratory sensitization classification (European Chemicals Agency, 2024). No exposure measurements were conducted during the index SICs; only time-weighted averages from similar SICs were available, and RAC acknowledges that real-time and peak exposures—critical for verifying that challenge atmospheres remained below irritant thresholds—were not captured, preventing confirmation that observed responses reflect sensitization rather than irritation (European Chemicals Agency, 2024; Pemberton and Kimber, 2022).

Even among the four cases considered potentially relevant following clarifications, core uncertainties remain: missing exposure measurements and incomplete clinical histories (e.g., atopy, non-specific bronchial hyperresponsiveness, baseline asthma status) (European Chemicals Agency, 2024; Pemberton and Kimber, 2022). These factors directly affect whether clinical reactions can be plausibly interpreted as immunologically mediated, as required by CLP Annex I expectations for well-documented human evidence (European Union, 2008). Importantly, this critique does not dispute that workers in MMA-using environments can develop respiratory symptoms; rather, it concerns whether the cited SIC evidence is sufficiently specific and well-controlled to support “respiratory sensitization” as the causal interpretation (Pemberton and Kimber, 2022).

The dossier context also includes sixteen negative SICs conducted with only or predominantly MMA, including individuals investigated for suspected MMA-related disease (European Chemicals Agency, 2024). The majority of these negative challenges originated from experienced centres participating in the Suojalehto et al. multicentre cohort and were performed using established SIC protocols consistent with international clinical practice and designed to avoid overtly irritating exposure conditions. These negative SIC data were not published in the scientific literature but were assessed by RAC in the course of its review (European Chemicals Agency, 2024). While the conduct of these SICs supports their overall quality and interpretability, negative SIC outcomes cannot exclude respiratory sensitization with certainty. False-negative results may arise from factors such as inter-individual disease variability, timing of testing relative to active disease, limited exposure duration during challenge, or clinical remission at the time of testing. RAC therefore considered the negative SICs as relevant contextual information when interpreting a limited number of positive findings, rather than as definitive evidence against respiratory sensitization (European Chemicals Agency, 2024).

## 3 The clinical literature: challenges in causal attribution

Frequently cited clinical publications contribute valuable occupational asthma phenotyping and surveillance, but they do not consistently isolate MMA as the unique causative agent under conditions that allow a respiratory sensitization conclusion. The published Suojalehto cohort does not contain substance-specific SICs for MMA; although SIC-positive reactions were reported within an acrylate-exposed population, attribution to MMA cannot be distinguished from typical co-exposures (Suojalehto et al., 2020), and only the unpublished Annex 5 case files attempt substance-specific evaluation (European Chemicals Agency, 2024). In the surveillance-based series by Walters et al. (2017), MMA exposure occurred in workers with suspected occupational asthma, but SIC positivity was limited, and some cases involved exposure to complex systems where other sensitizers may have been present (Walters et al., 2017). Across these studies, exposure monitoring and detailed peak characterization are limited, and clinical documentation may not always allow a confident distinction between immune-mediated sensitization and irritation-triggered responses (Pemberton and Kimber, 2022; Suojalehto et al., 2020; Walters et al., 2017).

This literature therefore supports a cautious interpretation: while respiratory symptoms and asthma-like presentations occur in MMA-relevant workplaces, the currently cited clinical evidence does not consistently meet the methodological and documentation features typically needed to infer MMA-specific respiratory sensitization with high confidence (European Chemicals Agency, 2024; Pemberton and Kimber, 2022; Suojalehto et al., 2020; Walters et al., 2017).

## 4 NAMs and mechanistic plausibility: a coherent alternative explanation

In contrast to heterogeneous clinical reports, mechanistic evidence from NAMs has been interpreted as consistently negative for key early events associated with respiratory sensitization pathways. MMA shows negligible peptide/protein reactivity—demonstrated in a refined *in chemico* amino-reactivity assay. The Gly-pNA chemoassay, a DPRA-like peptide depletion method—indicating no meaningful engagement of Key Event 1 (KE 1) (Simoneit et al., 2025). Likewise, no epithelial danger signaling (KE 2) has been observed in ALIsens® 3D alveolar coculture systems or rat precision-cut lung slices (PCLS), where MMA fails to induce cytokines IL-1α, GM-CSF, GRO-α, IFN-γ, IL-6, MIP-1β and the chemokine (MIP-3α) (Hess et al., 2016; Gutleb et al., 2026). Furthermore, KE 3 is unsupported, as MMA does not activate dendritic-like THP-1 cells in ALIsens®, showing no upregulation of TSLPr, even at maximal achievable exposures (Gutleb et al., 2026).

While NAMs are not a substitute for well-documented human evidence and are not yet accepted as standalone tools for regulatory classification, they are increasingly recognized as valuable complementary sources of information. Current regulatory frameworks, including CLP, continue to prioritize human data; however, NAMs can provide critical insight into mechanistic plausibility, particularly when human evidence is sparse, methodologically constrained, or difficult to interpret. In the case of MMA, the concordant absence of key sensitization-related events across multiple NAM platforms does not replace clinical evidence, but it does offer a biologically coherent context for interpreting whether reported respiratory reactions align more closely with immune-mediated sensitization or with irritation-driven mechanisms (Faulhammer et al., 2026).

Because KEs 4 (Th2 cell activation) and 5 (allergic respiratory hypersensitivity) depend on the successful progression of KE1–KE3 (i.e., antigen formation, epithelial danger signaling, and dendritic cell activation), their activation is biologically implausible when upstream events remain negative (Faulhammer et al., 2026). Notably, this mechanistic coherence becomes particularly relevant when the human evidence stream is limited by insufficient exposure control, incomplete documentation, or unclear substance specificity. In such cases, mechanistic plausibility should not be treated as ancillary; it can serve as an anchor for interpreting whether observed human reactions align with sensitization biology or are more plausibly explained by irritation (Faulhammer et al., 2026; Pemberton and Kimber, 2022).

RAC’s 2024 opinion explicitly acknowledges uncertainties related to exposure characterization and substance specificity in the available SIC data but concludes that these uncertainties do not preclude classification under a precautionary interpretation of CLP. These considerations align with recent analyses highlighting the inherent limitations of clinical elicitation data for low molecular weight chemicals, where incomplete exposure characterization, co-exposures, and insufficient mechanistic resolution can lead to uncertainty or misclassification when used in isolation for regulatory hazard identification (Scheel et al., 2025). Making such decision rationales more explicit—and systematically integrating mechanistic evidence alongside human data—could improve transparency and facilitate scientific dialogue around borderline classifications.

Across KE1–KE3, MMA is consistently negative (Gly-pNA amino-reactivity; rat PCLS IL-1α; ALIsens® dendritic-cell activation/cytokines), while acknowledging that no validated NAM battery or OECD Test Guidelines currently exist for respiratory sensitization.

Further generation of mechanistically focused NAM data—particularly gene-based analyses in human precision-cut lung slices (hPCLS) targeting epithelial danger signaling, innate immune activation, and dendritic-cell–relevant pathways (e.g., cytokines, chemokines, antigen-presentation and co-stimulatory markers aligned with KE2–KE3)—could further inform the interpretability and biological plausibility of the existing evidence base and, if consistently negative, strengthen the scientific assessment of whether MMA warrants classification as a respiratory sensitizer.

## 5 Precaution with standards: strengthening interpretability without weakening protection

The MMA case raises a fundamental point about the precautionary principle in classification: precaution is essential, but it should be applied using “minimum evidence standards” that ensure interpretability and transparency. CLP Annex I already sets expectations for human evidence to be well documented, substance-specific and not confounded by irritating concentrations with an emphasis on relevance and reliability (European Union, 2008). When SIC data lack real-time exposure measurements, controlled non-irritant conditions cannot be verified, and causal interpretation becomes uncertain—particularly for substances with known irritant properties (Pemberton and Kimber, 2022). Importantly, this framework is not specific to MMA but is equally relevant for other low-molecular-weight chemicals and workplace substances for which limited or poorly controlled human elicitation data coexist with negative or equivocal mechanistic evidence, including respiratory irritants, weakly protein-reactive chemicals, or complex exposure mixtures.

Rather than treating SIC results as inherently decisive, regulatory practice could benefit from explicit minimum requirements for SIC use in hazard classification, including:

1. Substance-specific challenge atmospheres, or fully characterized mixtures where co-exposures are unavoidable;
2. Real-time exposure monitoring, including peak capture and documentation of exposure stability;
3. Complete clinical characterization, including atopy status, baseline asthma diagnosis, and non-specific bronchial hyperresponsiveness;
4. Transparent reporting of SIC protocols and (where feasible) raw exposure data;
5. Explicit integration of NAM/AOP-based mechanistic evidence to support or challenge sensitization plausibility (if available) (European Chemicals Agency, 2024; European Union, 2008; Faulhammer et al., 2026; Pemberton and Kimber, 2022).

Such standards would not reduce precaution. They would strengthen it by ensuring that classifications are grounded in evidence that is reproducible, interpretable, and mechanistically coherent—especially as NAMs become more widely used to support regulatory decisions (Faulhammer et al., 2026).

## 6 Discussion

MMA’s proposed classification as a respiratory sensitizer illustrates a broader regulatory challenge: how to reconcile limited and variably documented human evidence with increasingly informative mechanistic data from NAMs. The NAM evidence discussed here became available after the 2024 RAC opinion and therefore could not have been considered in that assessment; it is presented here to inform ongoing and future scientific interpretation and dialogue. The concerns raised in this Opinion are not intended to challenge precautionary regulation, but to argue that precaution is most robust when applied in a transparent, reproducible, and scientifically coherent manner.

The SIC cases cited in support of MMA sensitization provide important clinical observations, yet their interpretability is constrained by limited exposure characterization, uncertainties in substance specificity, and incomplete clinical documentation. These limitations are particularly consequential for a substance with well-established irritant properties, where non-specific bronchoconstriction represents a plausible alternative explanation (European Chemicals Agency, 2024; Pemberton and Kimber, 2022). In this context, the presence of multiple negative SIC outcomes and long-standing use experience further underscores the uncertainty associated with drawing a definitive sensitization conclusion from a small and methodologically constrained positive SIC dataset.

Mechanistic evidence from NAMs offers a complementary perspective, showing a consistent absence of key early events associated with respiratory sensitization for MMA and aligning more closely with an irritant mode of action (Faulhammer et al., 2026). While NAMs cannot replace well-documented human evidence and negative findings cannot exclude sensitization with absolute certainty, they provide valuable insight into mechanistic plausibility, particularly when human data are difficult to interpret due to methodological limitations.

The practical implication is therefore not to weaken protection, but to refine how precaution is operationalized— *“precaution with standards.”* Establishing explicit minimum requirements for SIC interpretability and integrating NAM/AOP-based evidence transparently would strengthen scientific coherence and regulatory credibility. Applied to MMA, such an approach supports a balanced assessment that remains protective while avoiding classifications that are not strongly supported by interpretable and mechanistically plausible evidence. More broadly, aligning human evidence quality criteria with advancing mechanistic understanding is likely to become increasingly important as regulatory toxicology continues to transition toward non-animal, mechanism-centered decision frameworks (Faulhammer et al., 2026).
